# HTLV-1 and HTLV-2 infections significantly alter small RNA expression in asymptomatic carriers

**DOI:** 10.3389/fmed.2025.1547712

**Published:** 2025-02-17

**Authors:** Lorena Abreu Fernandes, Victor Ângelo Folgosi, Rodrigo Pessôa, Tatiane Assone, Jefferson Russo Victor, Jorge Casseb, Augusto César Penalva de Oliveira, Youko Nukui, Alberto José da Silva Duarte, Sabri Saeed Sanabani

**Affiliations:** ^1^Post-Graduation Program in Translational Medicine, Department of Medicine, Federal University of São Paulo, São Paulo, Brazil; ^2^Laboratory of Medical Investigation LIM-56, Division of Dermatology, University of São Paulo Medical School, São Paulo, Brazil; ^3^Department of Neurology, Institute of Infectology Emílio Ribas (IIER), São Paulo, Brazil; ^4^Department of Hematology, Faculty of Medicine, University of São Paulo, São Paulo, Brazil; ^5^Laboratory of Medical Investigation 03 (LIM03), Clinics Hospital, Faculty of Medicine, University of São Paulo, São Paulo, Brazil

**Keywords:** HTLV-1, HTLV-2, sRNA expression, Gene Ontology, KEGG pathways, miRNAs

## Abstract

**Introduction:**

This study investigates the impact of HTLV-2 infection on small RNA (sRNA) expression profiles, building on previous findings related to HTLV-1.

**Methods:**

Using Illumina Massive Parallel Sequencing, we analyzed sRNA profiles from asymptomatic HTLV-1 and HTLV-2 infected individuals and healthy controls.

**Results:**

Our results reveal significant differential expression of 331 known and 441 novel sRNAs among the groups, including miRNAs, piRNAs, and tRNAs. Notably, distinct clusters of sRNA expression patterns were identified, with specific miRNAs showing significant upregulation or downregulation in HTLV-1 and HTLV-2 infections. Gene Ontology analysis indicated significant involvement of target genes in transcription regulation and RNA-binding processes, while KEGG pathway analysis highlighted enrichment in cancer-related pathways and signaling cascades such as FoxO, Ras, and MAPK. Network analysis identified key miRNAs, such as hsa-miR-20b-5p and hsa-let-7e-5p, as central regulators with extensive interactions, suggesting their potential role in the pathogenesis and immune response of HTLV infections.

**Discussion:**

These findings provide a foundation for future research into the molecular mechanisms of HTLV infections and the development of targeted therapies. The identified sRNAs, especially important miRNAs such as hsa-miR-20b-5p and hsa-let-7e-5p, could serve as potential biomarkers for disease progression or as therapeutic targets to modulate the immune response and disrupt viral pathogenesis. This opens up new avenues for precision medicine in HTLV-associated diseases.

## Introduction

Human T-lymphotropic viruses type 1 and type 2 (HTLV-1 and HTLV-2) are closely related delta retroviruses with a shared affinity for T-lymphocytes. Unlike HIV, HTLVs are rarely found in cell-free plasma and exhibit limited replication in infected individuals (1). HTLV-1 is globally distributed, with endemic hotspots in southern Japan, the Caribbean, parts of South America, Africa, the Middle East, and Melanesia (2, 3). HTLV-2, in contrast, is primarily endemic among Amerindian populations in the Americas and African Pygmies (4–6). It is estimated that 15 to 20 million people worldwide are infected with HTLV, with 670,000 to 890,000 infected with HTLV-2 (7–9). Transmission occurs through parenteral exposure, sexual contact, and vertical transmission during pregnancy and breastfeeding (10).

HTLV-1 is associated with diseases such as adult T-cell leukemia/lymphoma (ATL) and HTLV-associated myelopathy/tropical spastic paraparesis (HAM/TSP), as well as inflammatory conditions like lymphocytic pneumonitis and uveitis (11–15). Although HTLV-2 is less pathogenic, it has been linked to HAM/TSP and an increased incidence of infections like acute bronchitis and pneumonia, suggesting a subtle immunomodulatory role (10, 16).

ATL manifests in a small percentage of infected individuals after a latent period, characterized by malignant lymphocytosis and systemic symptoms (17, 18). HAM/TSP affects approximately 2% of those infected with HTLV-1 or HTLV-2, presenting with progressive neurological symptoms, including spastic paraparesis and sensory disturbances (19).

Small RNAs (sRNAs), including microRNAs (miRNAs), are crucial regulators of gene expression and play significant roles in cellular processes such as development, differentiation, and immune response (20). MicroRNAs, although constituting only 1–5% of the human genome, regulate over 30% of protein-coding genes (21). Alterations in sRNA expression are implicated in the pathogenesis of viral infections (22), with viral infections causing significant changes in host sRNA expression profiles, potentially influencing disease progression or asymptomatic states (23–27).

Our previous study examined the global noncoding RNAome expression profile in HAM patients, asymptomatic HTLV-1-infected carriers, and healthy controls (HC) (28). We identified a distinct cluster of 38 sRNA genes with elevated expression in HC, underscoring the significant impact of HTLV-1 on the sRNA landscape. Notably, miRNAs such as hsa-miR-183, 451a, and 144 were particularly affected, suggesting their potential role in the host’s response to HTLV-1 infection.

Given these findings, we aim to investigate whether HTLV-2 infection similarly alters sRNA expression. Despite its lower pathogenicity, HTLV-2 shares many biological characteristics with HTLV-1, making it a compelling subject for comparative studies (29). This pilot study expands our investigation to include small RNA profiles from six patients with confirmed HTLV-2 infection, aiming to provide a comprehensive understanding of how different HTLV types impact the cellular noncoding RNAome.

## Materials and methods

### Enrollment of patients and preparation of samples

At the time of blood donation, six individuals were identified as HTLV-2 carriers. Following approval from the local Ethics Committee and in accordance with ethical guidelines, these individuals provided written informed consent for their blood samples to be included in this study. The diagnosis of HTLV-2 infection was conducted using enzyme immunoassays: Murex HTLV I + II (Abbott/Murex, Wiesbaden, Germany) and Vironostika HTLV-I/II (BioMérieux bv, Boxtel, The Netherlands). Confirmation of HTLV-2 infection was achieved through Western blot and PCR, utilizing the HTLV BLOT 2.4 assay (Genelabs Diagnostics, Science Park, Singapore) at the laboratory of the blood bank. Peripheral Blood Mononuclear Cells (PBMCs) were isolated using Ficoll–Hypaque density centrifugation (Amersham, Upsala, Sweden) and subsequently stored as previously described (28, 30). For the HTLV-1 infected asymptomatic subjects (*n* = 10) and HC (*n* = 6), small RNA NGS sequences were retrieved from the Zenodo repository database. Detailed procedures for the recruitment and sample preparation of these groups have been described in a recent study by our group (28).

### Small RNA extraction and sequencing

Total RNA and small RNA (sRNA) were extracted using the MiRNeasy Mini Kit (Qiagen, Hilden, Germany) and TRIzol (Life Technologies, United States) protocols, respectively, as previously published (30). For sRNA sequencing, libraries were prepared using the TruSeq sRNA Sample Preparation Kit (Illumina, San Diego, CA, United States), and sequencing on the MiSeq platform were performed according to the manufacturer’s instructions (Illumina, San Diego, CA, United States) and a previous protocol (28, 30). Libraries from all samples were pooled, and 8–10 pM of the pool was sequenced on the Illumina MiSeq platform as per the manufacturer’s protocol.

### sRNA data analysis and interpretation

The analysis of sRNA data began with base-calling, demultiplexing, and the generation of trimmed FASTQ files using MiSeq Reporter versions 2.3 and 2.4.1.3 (Illumina, Inc.). Only high-quality reads, defined as those with a Sanger score greater than 30, were selected for further analysis. These reads were aligned to the hg19 whole genome build using Strand NGS version 3.1 (Strand Life Science, Bengaluru, India), leveraging default parameters for the sRNA alignment and analysis pipeline. For sequence annotation and differential expression analysis of known and novel sRNA, as well as mature miRNAs, Strand NGS’s default algorithms were employed. Novel sRNAs were identified and classified using a decision tree method with 3-fold validation accuracy, following the model described by Langenberger et al. (31). Distributions of sRNA data across all conditions were normalized using the quantile normalization algorithm, with the median value of all samples set as the baseline transformation. To qualify as novel or known sRNA for further analyses, sequences had to meet a minimum read coverage criterion of greater than 5. The Shapiro–Wilk method was employed to test for normality in the distribution of clean data within each group, with only those meeting a Shapiro–Wilk *p*-value greater than 0.95 considered for additional analysis. The adjusted *p*-value using Benjamini and Hochberg (BH) false discovery rate (FDR) method by default was applied to correct for the occurrence of false positive results. Differential expression was determined for sRNAs exhibiting fold changes greater than 10.0 and fold change >10 and FDR with a *p* (corr) cut-off ≤0.005.

The comparative analysis of differential expression was conducted among three groups: HTLV-1, HTLV-2, and HC, forming the following pairs: HTLV-1 vs. HTLV-2, HTLV-1 vs. HC, and HTLV-2 vs. HC. Visualization of the expression data was achieved through heatmap and volcano plots, facilitating the identification of significant differential expression patterns across the groups.

### Function enrichment and pathway network analysis

To explore the functional roles of the miRNAs, their putative target genes were predicted using TargetScan, miRDB, and miRTarBase, integrated within miRWalk v3 (updated August 2024) (32). These predicted genes underwent Gene Ontology (GO) enrichment analysis to identify associated biological processes, cellular components, and molecular functions. Additionally, the genes were classified according to the Kyoto Encyclopedia of Genes and Genomes (KEGG) pathways. Both GO and KEGG analyses were conducted using the DAVID database (33). Subsequently, Cytoscape software^1^ was employed for visual exploration and to investigate potential interactions between target miRNAs and mRNAs.

## Results

### Differential analysis of expression of known sRNA between groups

**Figure 1:**
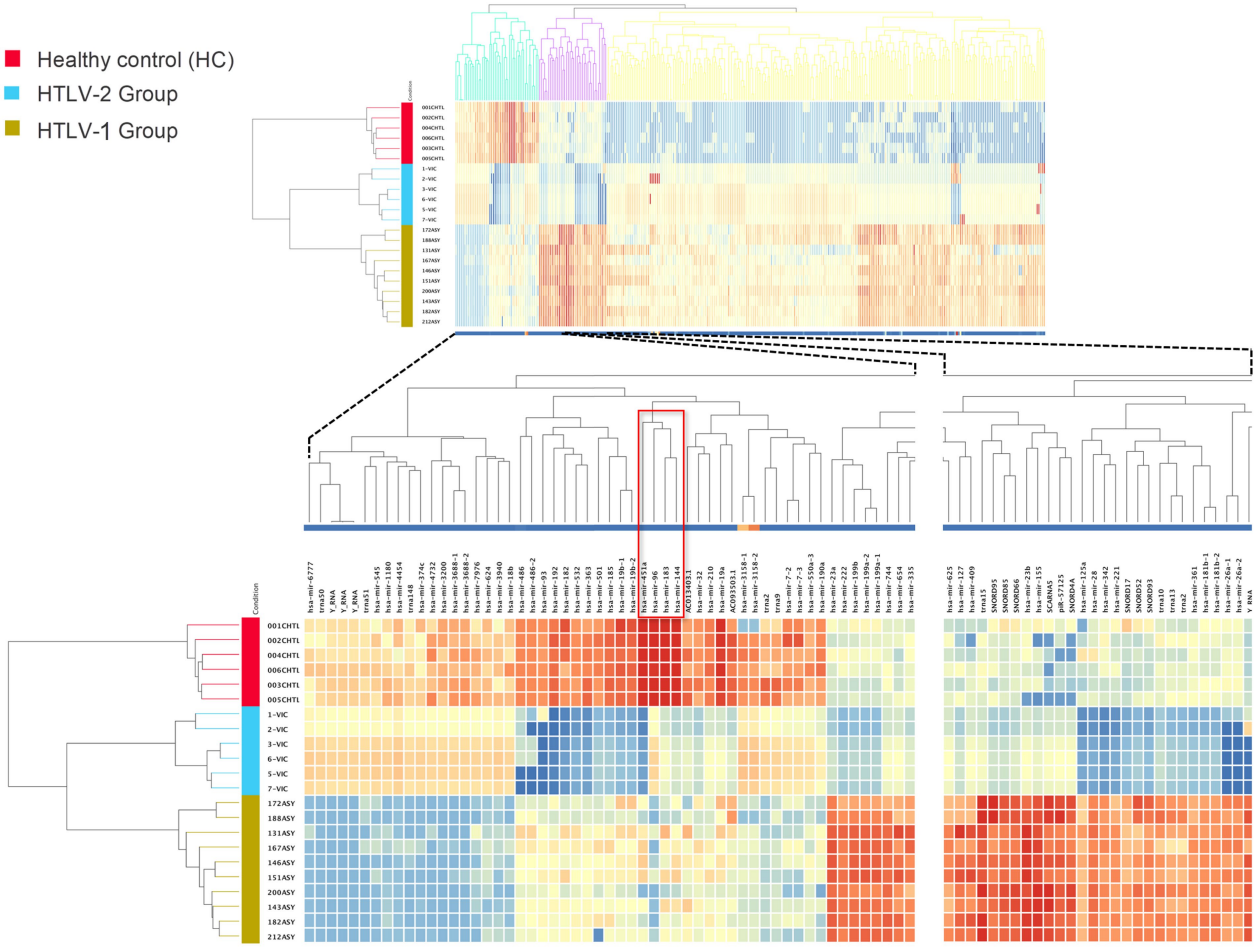
Heatmap of known small RNA expression profiles in healthy controls (HC), HTLV-2, and HTLV-1 groups. The dendrograms represent hierarchical clustering of samples and known small RNAs, highlighting distinct expression patterns across the groups. Red, blue, and yellow colors indicate the HC, HTLV-2, and HTLV-1 groups, respectively. The lower section provides a detailed view, highlighting miRNAs hsa-miR-183, hsa-miR-451a, and hsa-miR-144, which are highly expressed in HC compared to HTLV-1 and HTLV-2 groups. These miRNAs, indicated by a red empty box, motivated the current study. The intensity of the colors in the heatmap corresponds to the level of small RNA expression, with red representing high expression and blue representing low expression.

The differential expression analysis of known small RNAs (sRNAs) among asymptomatic HTLV-1 infected individuals, asymptomatic HTLV-2 infected individuals, and HC revealed significant expression profile differences. Using a stringent statistical approach (one-way ANOVA with Benjamini–Hochberg correction, *p*-value ≤0.001, fold change = 10), 331 known sRNAs were significantly differentiated between the three groups (Figure 1). These included 129 miRNAs, 3 piRNAs, 15 scRNAs, 3 scRNA pseudogenes, 12 snRNAs, 41 tRNAs, and 1 tRNA pseudogene (Supplementary Table S1). The heatmap visualization highlighted three distinct clusters of sRNA expression patterns. One notable cluster comprised 48 known sRNAs significantly upregulated in HC compared to both HTLV-1 and HTLV-2 infected individuals.

**Figure 2:**
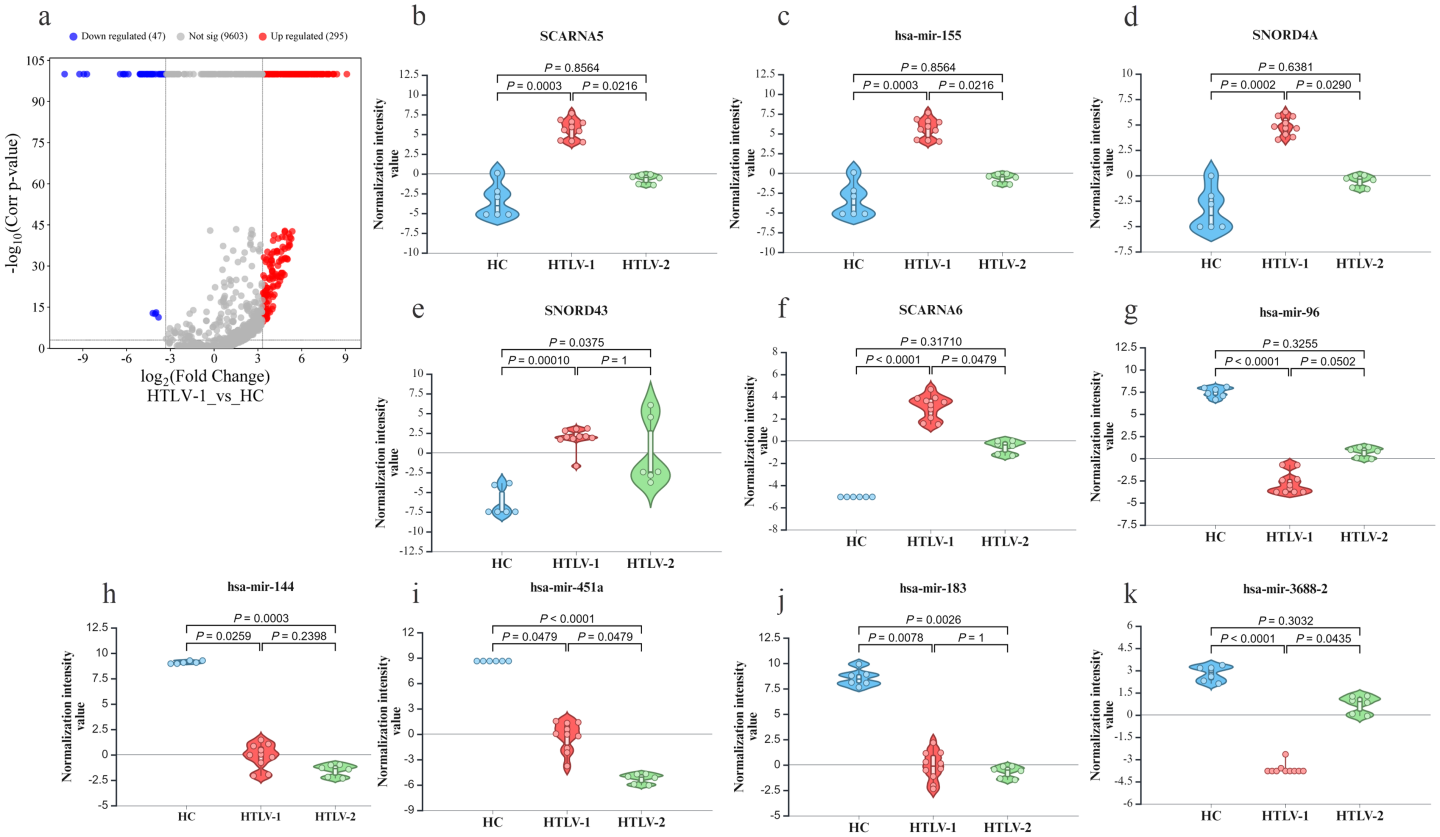
Differential expression of known small RNA (sRNA) profiles in healthy controls (HC), HTLV-2, and HTLV-1 groups. **(A)** Volcano plot showing differential expression of small RNAs between HTLV-1 and HC, with upregulated (red) and downregulated (blue) RNAs. **(B–K)** Violin plots displaying normalization intensity values for the top 5 most upregulated known sRNAs in HTLV-1 vs. HC (SCARNA5, hsa-miR-155, SNORD4A, SNORD43, SCARNA6) and the top 5 most downregulated (hsa-miR-96, hsa-miR-144, hsa-miR-451a, hsa-miR-183, hsa-miR-3688-2) across HC, HTLV-1, and HTLV-2 groups. Significant differences are indicated by *p*-values.

**Figure 3:**
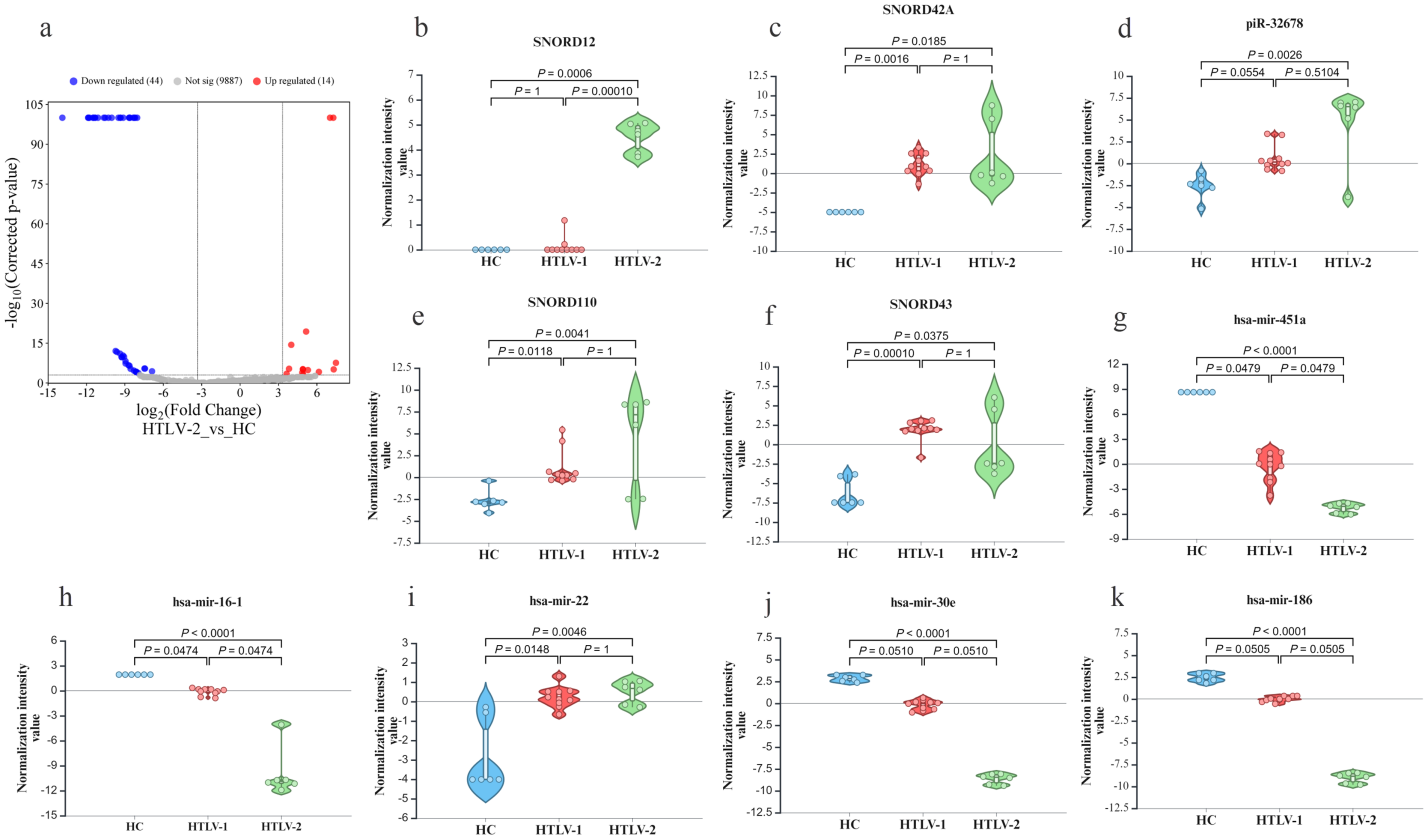
Differential expression of known small RNA (sRNA) profiles in healthy controls (HC), HTLV-2, and HTLV-1 groups. **(A)** Volcano plot showing differential expression of small RNAs between HTLV-2 and HC, with upregulated (red) and downregulated (blue) RNAs. **(B–F)** Violin plots display normalization intensity values for the top 5 most upregulated known sRNAs in HTLV-1 vs. HC (SCARNA5, hsa-miR-155, SNORD4A, SNORD43, SCARNA6). **(G–K)** Violin plots show the top 5 most downregulated sRNAs (hsa-miR-96, hsa-miR-144, hsa-miR-451a, hsa-miR-183, hsa-miR-3688-2) across HC, HTLV-1, and HTLV-2 groups. Significant differences are indicated by *p*-values.

In comparisons between HTLV-1 infected individuals and HC, 47 sRNAs were downregulated, while 295 were upregulated (Figure 2A). Among the top five most upregulated and downregulated sRNAs, six were miRNAs (Figures 2B–K). SCARNA5 was the most upregulated with a fold change greater than 545, followed by hsa-miR-155 and SNORD4A. Conversely, hsa-miR-96 was the most downregulated with a fold change exceeding −1,211, followed by hsa-miR-144 and 451a. In the HTLV-2 group, 14 sRNAs were upregulated and 44 were downregulated compared to HC (Figure 3A). No miRNA was among the top five upregulated sRNAs, but all five-top downregulated sRNAs were miRNAs (Figures 3B–K). The top five upregulated sRNAs in HTLV-2 individuals, except piR-32678, were SNORNA types, with SNORD12 and SNORD42A showing fold changes greater than 197 and 158, respectively. Conversely, hsa-miR-451a and hsa-miR-16-1 were the most downregulated, with fold changes exceeding −15,158 and − 3,741. Notably, SNORD43 and hsa-miR-451a showed similar expression patterns in both HTLV-1 and HTLV-2 groups (Figures 2E,I).

The Venn diagram in Supplementary Figure S1A indicates no common sRNAs across all four lists in both HTLV-1 and HTLV-2 groups. However, specific overlaps exist; all significantly upregulated sRNAs in the HTLV-2 group (entity list 1), except piR-32678, were also upregulated in the HTLV-1 group (entity list 3). Two sRNAs, hsa-miR-26a-1 and hsa-miR-26a-2, upregulated in HTLV-1 (entity list 3), were downregulated in HTLV-2 (entity list 2). Notably, entity list 3 is distinct with 280 unique upregulated sRNAs, and entity list 2 has 31 unique entities, highlighting the predominance of unique sRNAs in these lists. The 3-D principal component analysis (PCA) of the 331 sRNA expression profiles revealed distinct clustering patterns among the three groups. The HTLV-1 infected group formed a separate cluster from the HTLV-2 infected group, with clear separation between them. Additionally, the HC samples clustered distinctly, showing strong separation from both HTLV-1 and HTLV-2 infected groups (Supplementary Figure S1B). This visualization demonstrates the unique sRNA expression signatures associated with each group, highlighting the molecular differences between HC, HTLV-1, and HTLV-2 infected individuals.

### Differential analysis of expression of novel sRNA between groups

We *de novo* employed a stringent statistical approach to analyze novel small RNAs (sRNAs) across the three groups, identifying 441 significantly differentiated sRNAs, including 45 miRNAs, 32 snoRNAs, 15 tRNAs, and 346 unknown sRNAs (Supplementary Table S2). The heatmap (Supplementary Figure S2) revealed two primary clusters: one with sRNAs upregulated in the HTLV-1 group compared to HC, and a second, more complex cluster divided into three subclusters. These subclusters included sRNAs upregulated in HC but downregulated in HTLV-1 without differential expression in HTLV-2, sRNAs underexpressed in HTLV-1, and sRNAs highly expressed in HTLV-2 but underexpressed in HTLV-1, with no differential expression in HC. These patterns offer insights into the distinct sRNA expression profiles in different groups, potentially shedding light on the molecular mechanisms of HTLV infections.

**Figure 4:**
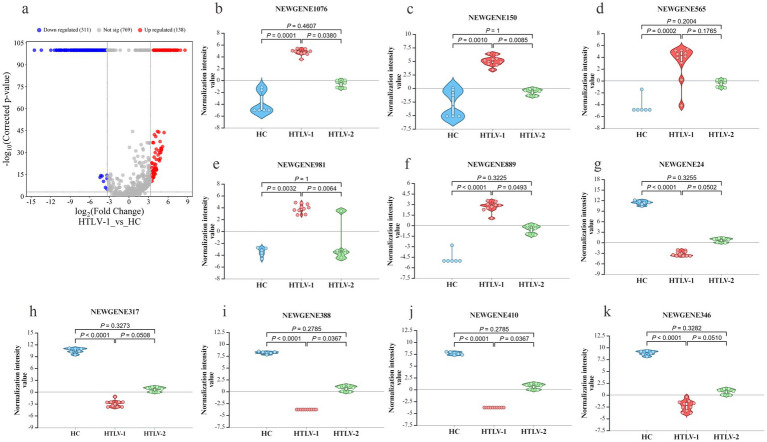
Differential expression of novel small RNA (sRNA) profiles in healthy controls (HC), HTLV-2, and HTLV-1 groups. **(A)** Volcano plot showing differential expression of novel sRNA between HTLV-1 and HC, with upregulated (red) and downregulated (blue) sRNA. **(B–F)** Violin plots display normalization intensity values for the top 5 most upregulated novel sRNAs in HTLV-1 vs. HC (NEWGENE1076, 150, 565, 981, AND 989). **(G–K)** Violin plots show the top 5 most downregulated sRNAs (NEWGENE24, 317, 388, 410, and 346) across HC, HTLV-1, and HTLV-2 groups. Significant differences are indicated by *p*-values.

**Figure 5:**
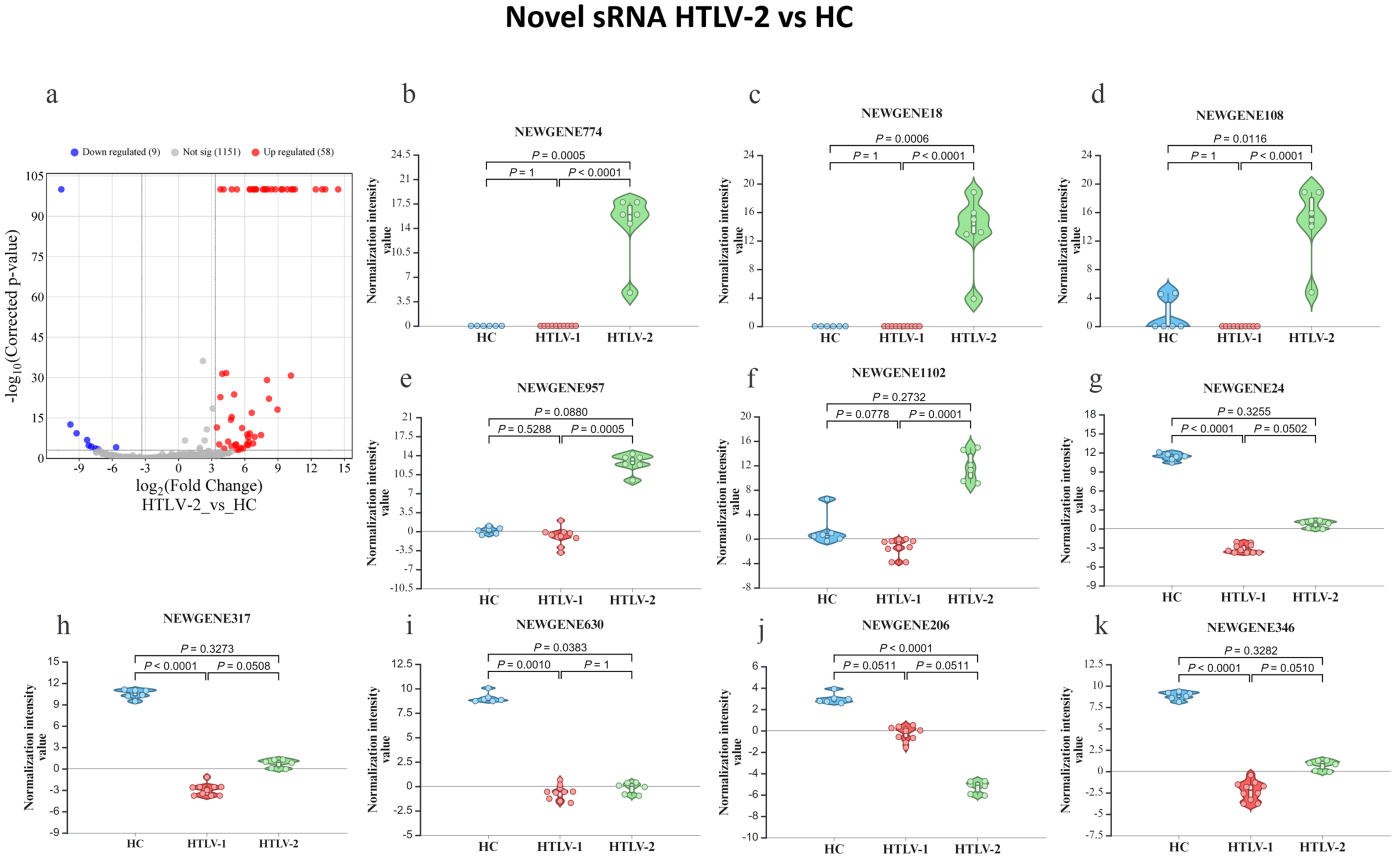
Differential expression of novel small RNA (sRNA) profiles in healthy controls (HC), HTLV-2, and HTLV-1 groups. **(A)** Volcano plot showing differential expression of novel sRNA between HTLV-2 and HC, with upregulated (red) and downregulated (blue) sRNA. **(B–F)** Violin plots display normalization intensity values for the top 5 most upregulated novel sRNAs in HTLV-2 vs. HC (NEWGENE774, 18, 108, 957, and 1,102). **(G–K)** Violin plots show the top 5 most downregulated sRNAs (NEWGENE24, 317, 383, 206, and 346) across HC, HTLV-1, and HTLV-2 groups. Significant differences are indicated by *p*-values.

In the HTLV-1 group versus HC, 449 novel sRNAs were significantly dysregulated, with 137 upregulated and 311 downregulated (Figure 4A). The most upregulated were NEWGENE1076, NEWGENE150, NEWGENE565, NEWGENE981, and NEWGENE889 (Figures 4B–F), while the most downregulated included NEWGENE24, NEWGENE317, NEWGENE388, NEWGENE410, and NEWGENE346 (Figures 4G–K), with NEWGENE981 being the only miRNA type. The HTLV-2 versus HC comparison revealed 58 upregulated and 9 downregulated novel sRNAs (Figure 5A), with the top upregulated being NEWGENE774, NEWGENE18, NEWGENE108, NEWGENE957, and NEWGENE1102 (Figures 5B–F). Notably, NEWGENE24, NEWGENE317, and NEWGENE346 were downregulated in both HTLV-1 and HTLV-2 groups compared to HC (Figures 5G–K). The Venn diagram (Supplementary Figure S3A) illustrates the overlap of regulated sRNAs among the four subgroups: HTLV-2 versus HC (upregulated, entity list 1; downregulated, entity list 2) and HTLV-1 versus HC (upregulated, entity list 3; downregulated, entity list 4). No entities are common to all lists, but overlaps include seven entities between lists 2 and 4, and twelve between lists 1 and 3. Entity list 2 has 289 unique downregulated sRNAs, and list 1 has 126 unique upregulated sRNAs, highlighting distinct sRNA profiles. Additionally, 15 entities are upregulated in list 3 while downregulated in list 2, indicating opposite expression patterns in HTLV-1 and HTLV-2 infections compared to HC. We generated a 3D PCA plot based on the expression profiles of 449 significantly deregulated novel sRNAs. This analysis revealed distinct clustering patterns similar to those observed in the known sRNA data (Supplementary Figure S3B). The HC samples formed a separate cluster, clearly distinguishable from the HTLV-infected groups. Moreover, the HTLV-1 and HTLV-2 samples also demonstrated clear separation from each other, forming distinct clusters. This visualization underscores the unique expression patterns of novel sRNAs associated with each group, further highlighting the molecular distinctions between HC and individuals infected with HTLV-1 or HTLV-2.

### Differential analysis of expression of mature miRNAs between groups

**Figure 6:**
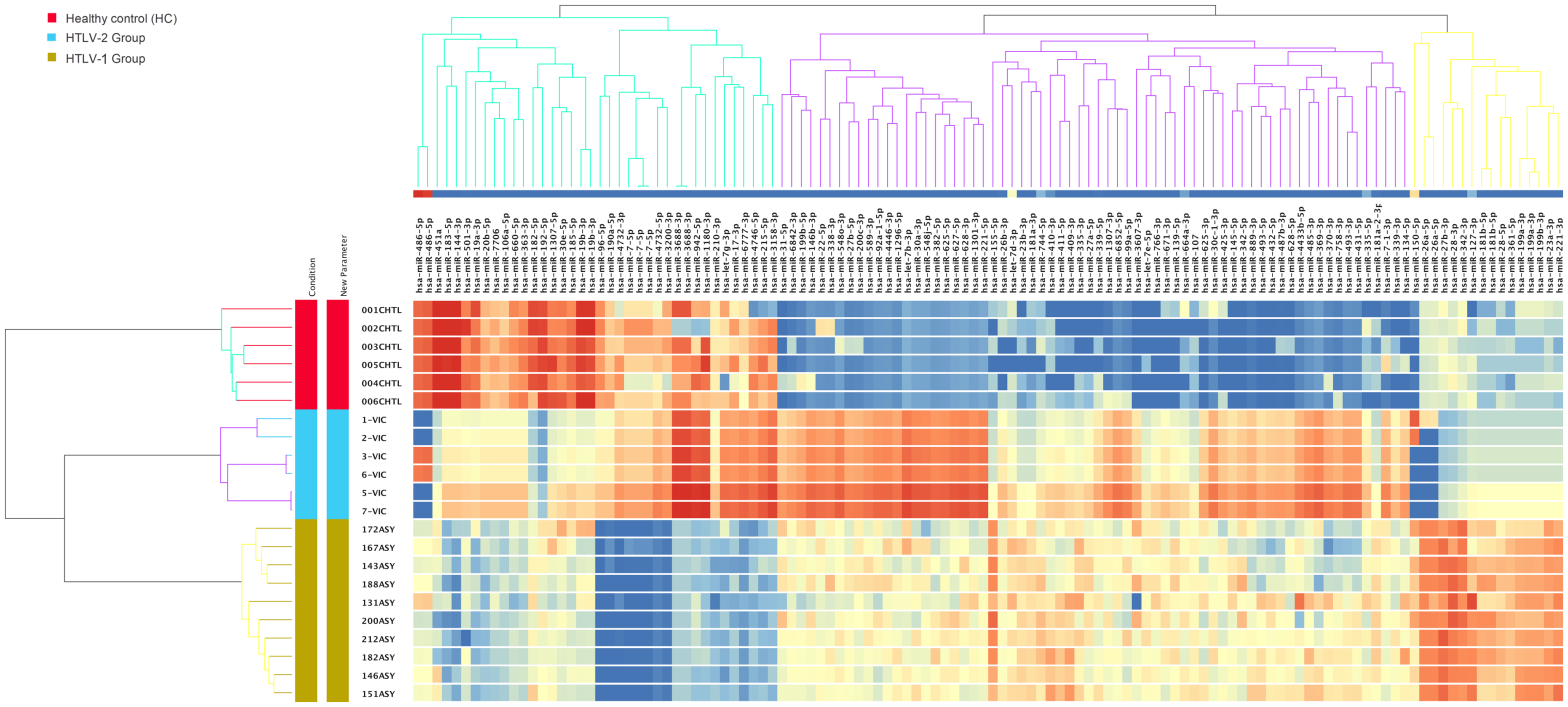
Heatmap of mature microRNA (miRNA) expression profiles in healthy controls (HC), HTLV-2, and HTLV-1 groups. The dendrograms represent hierarchical clustering of samples and miRNA, highlighting distinct expression patterns across the groups. Red, blue, and yellow colors indicate the HC, HTLV-2, and HTLV-1 groups, respectively. The intensity of the colors in the heatmap corresponds to the level of miRNA expression, with red representing high expression and blue representing low expression.

The replicative statistical analysis of mature miRNA revealed 120 active miRNAs significantly differentiated between the three groups (Supplementary Table S3). Three major clusters of these miRNAs were observed to differentiate the groups as shown in the heatmap depicted in Figure 6. The first cluster is composed of two main subclusters. Inspection of the first subcluster revealed three miRNAs (hsa-miR-451a, hsa-miR-183-5p, hsa-miR-144-3p) that were strongly upregulated in the HC group but strongly downregulated in HTLV-1 infected groups. Similarly, in the second subcluster, six miRNAs (hsa-miR-96-5p, hsa-miR-190a-5p, hsa-miR-4732-3p, hsa-miR-7-5p, hsa-miR-4732-5p, and hsa-miR-3200-3p) were strongly downregulated in the HTLV-1 group when compared to both HTLV-2 and HC groups. The second major cluster consists of 66 miRNAs and formed three subclusters, with almost all of these miRNAs downregulated in HC when compared to the HTLV-1 and 2 infected groups. The first subcluster, which contains 22 miRNAs, demonstrates that all of them were only and strongly upregulated in HTLV-2. hsa-miR-155-5p is unique in subcluster two, which showed strong upregulation in HTLV-1 but downregulation in HTLV-2 and HC groups. The same results hold true for all 16 miRNAs that formed the third major cluster, with all these miRNAs showing strong upregulation in HTLV-1 but downregulation in HTLV-2 and HC groups.

**Figure 7:**
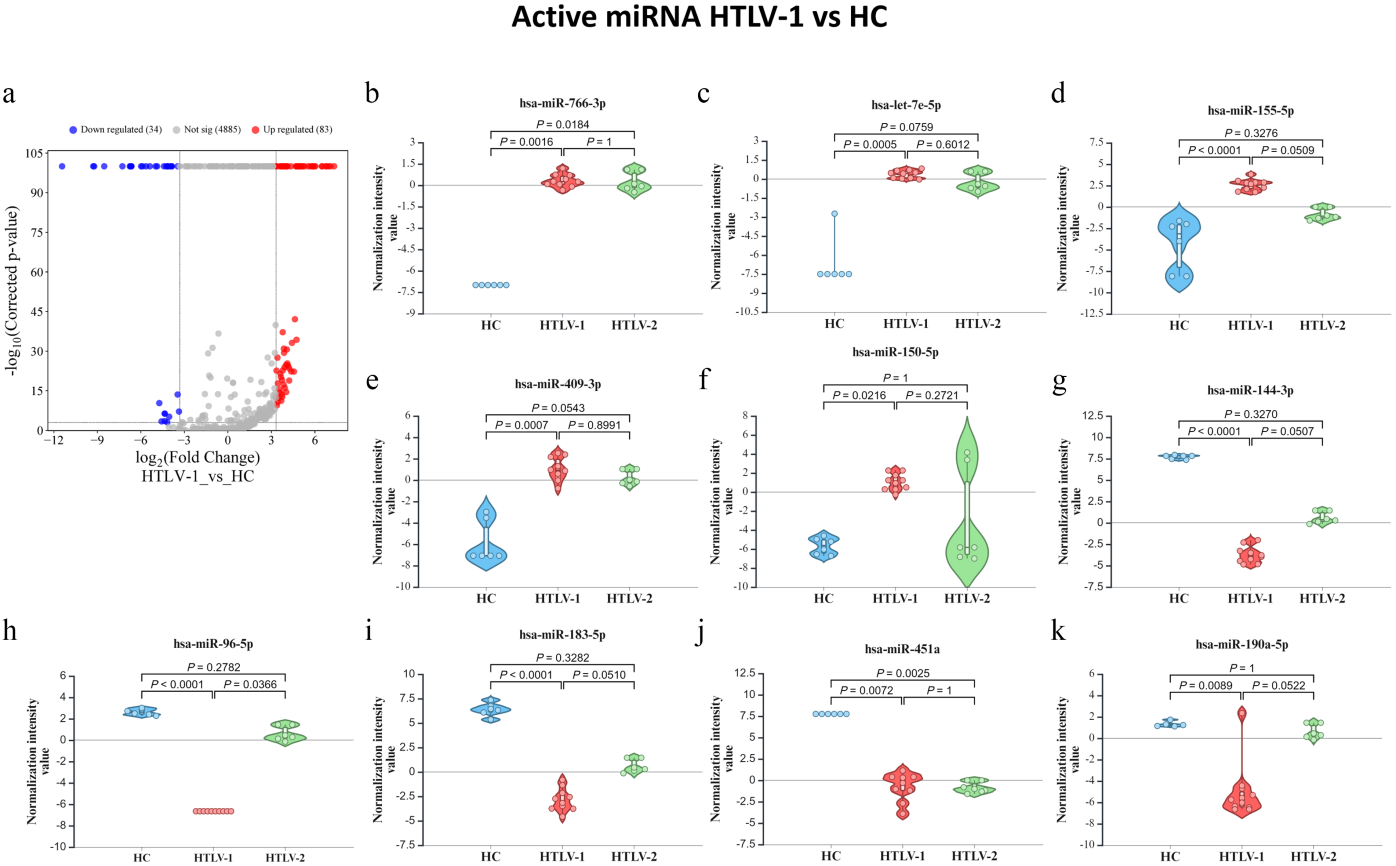
Differential expression of mature microRNA (miRNA) profiles in healthy controls (HC), HTLV-2, and HTLV-1 groups. **(A)** Volcano plot showing differential expression of novel miRNA between HTLV-1 and HC, with upregulated (red) and downregulated (blue) sRNA. **(B–F)** Violin plots display normalization intensity values for the top 5 most upregulated novel miRNAs in HTLV-1 vs. HC. **(G–K)** Violin plots show the top 5 most downregulated miRNAs across HC, HTLV-1, and HTLV-2 groups. Significant differences are indicated by *p*-values.

The differential expression analysis between HTLV-1 infected individuals and HC identified 83 upregulated and 34 downregulated mature miRNAs (Figure 7A). The top five upregulated miRNAs were hsa-miR-766-3p, hsa-let-7e-5p, hsa-miR-155-5p, hsa-miR-409-3p, and hsa-miR-150-5p (Figure 7B–F). In contrast, the most downregulated miRNAs included hsa-miR-144-3p, hsa-miR-96-5p, hsa-miR-183-5p, hsa-miR-451a, and hsa-miR-190a-5p (Figures 7G–K). For HTLV-2 versus HC, 20 miRNAs were significantly downregulated in the HTLV-2 infected group. The Venn diagram captures the overlap of significantly regulated miRNAs across three groups (Supplementary Figure S4A). Notably, two miRNAs, hsa-miR-26a-5p-1 (GI: MI0000083_1) and hsa-miR-26a-5p-2 (GI: MI0000750_2), are shared between entity lists 1 and 3, exhibiting opposite expression patterns in HTLV-1 and HTLV-2 infections compared to HC. Additionally, five miRNAs, hsa-miR-486-5p-1 (GI: MI0002470_2), hsa-miR-486-5p-2 (GI: MI0023622_1), hsa-miR-192-5p, hsa-miR-451a, and hsa-miR-144-3p overlap between entity lists 2 and 3. This diagram highlights distinct miRNA expression patterns, with HTLV-1 showing 81 unique upregulated and 29 downregulated miRNAs, while HTLV-2 has 13 unique downregulated miRNAs. PCA analysis of the 120 differentially expressed active miRNAs reveals distinct clustering patterns among the three groups. The HC patients form their own distinct cluster, while the HTLV-1 and HTLV-2 infected groups are also clearly separated from each other and from the HC group (Supplementary Figure S4B). This visual representation highlights the unique miRNA expression profiles of each group, demonstrating clear differences between HC, HTLV-1, and HTLV-2 infected individuals.

### Target genes, KEGG pathway, and GO enrichment analysis

**Figure 8:**
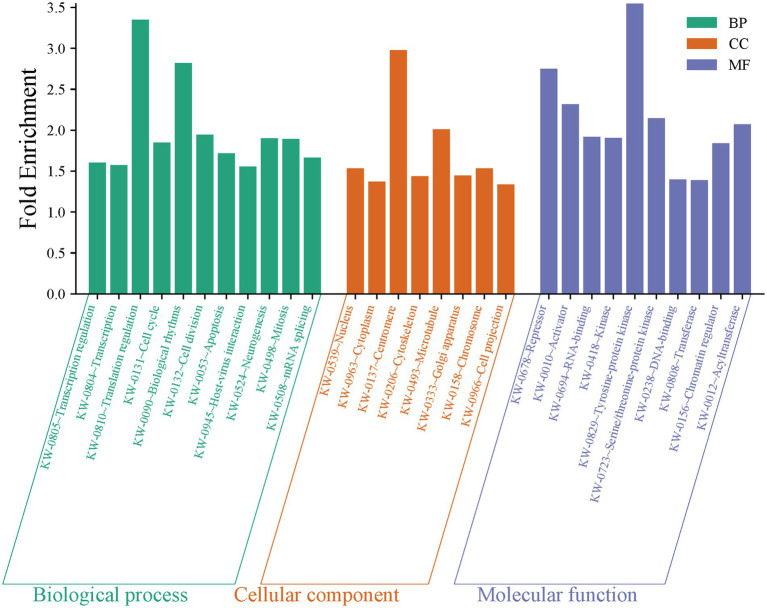
Gene set enrichment analysis of 120 active miRNAs significantly differentiated between HTLV-1, HTLV-2, and healthy controls (HC). The bar chart displays fold enrichment of gene ontology terms across three categories: biological process (BP), cellular component (CC), and molecular function (MF). Green bars represent BP terms, orange bars represent CC terms, and blue bars represent MF terms, highlighting key enriched terms such as transcription regulation, nucleus, and RNA-binding.

**Figure 9:**
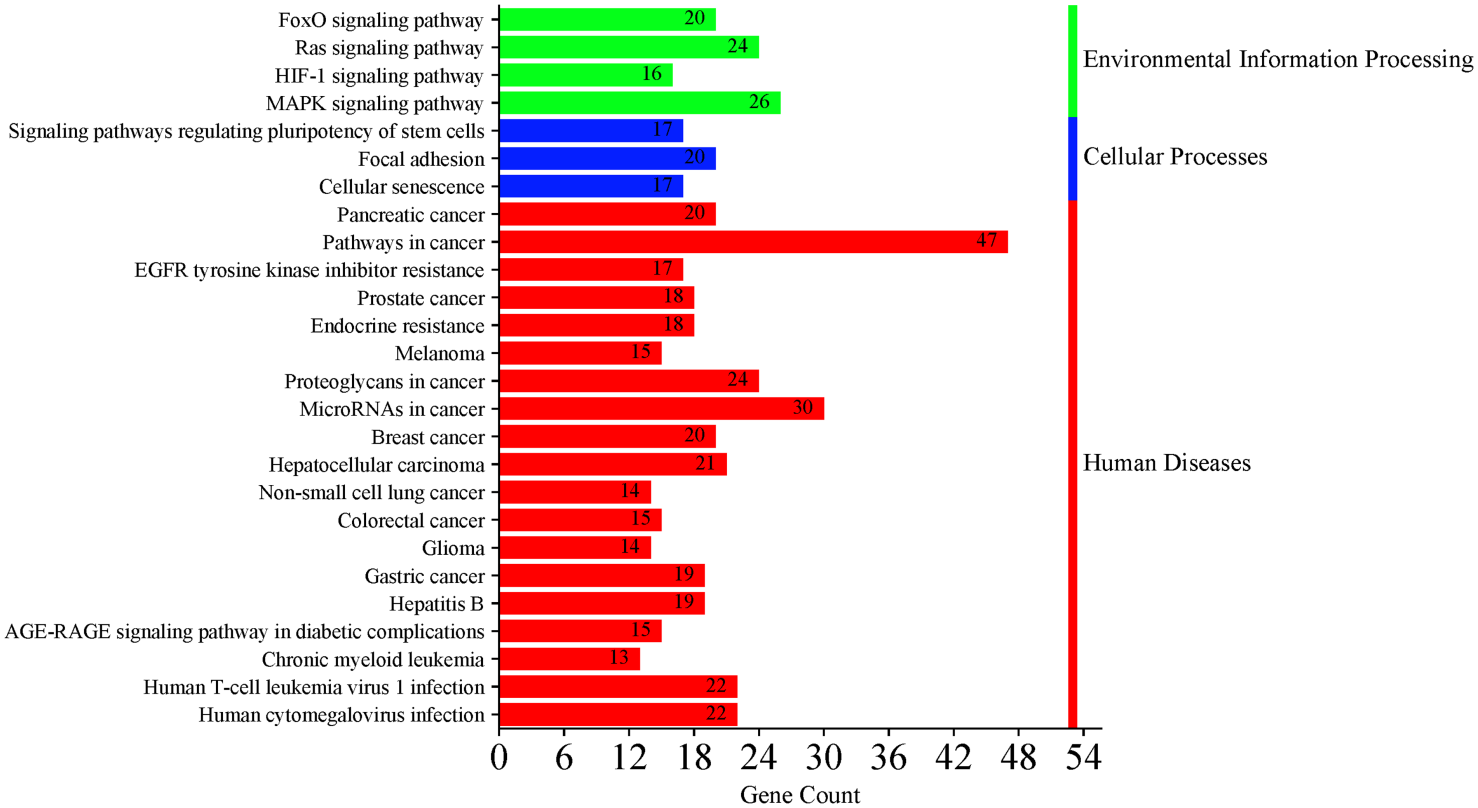
KEGG pathway analysis of predicted target genes from differentially expressed miRNAs in asymptomatic HTLV-1 and HTLV-2 carriers. The analysis shows significant enrichment in cancer-related pathways, signaling cascades (e.g., FoxO, Ras, MAPK), and viral infection processes. The top three enriched pathways are pathways in cancer, pancreatic cancer, and EGFR tyrosine kinase inhibitor resistance. Bar colors represent different categories: green for environmental information processing, blue for cellular processes, and red for human diseases.

Data from the 120 active miRNAs significantly differentiated between the three groups were used to predict miRNA target genes. This gene set enrichment analysis (GSEA) analysis by miRWalk v.3 resulted in 494 nonredundant target genes, as shown in Supplementary Table S4. Of these, 492 target genes were analyzed to determine the genes’ functional annotation by GO analysis using DAVID. The full record of remarkable functional annotations of target genes is provided in Supplementary Table S5. The pathways/terms identified were categorized into Uniprot (UP), KEGG pathway, and GO term enrichment analysis (GOTERM). Most target genes were involved in the phosphoprotein (KW-0597) post-translational modification (UP_KW_PTM). This finding is parallel with functional annotation of biological process (BP) by GO that includes the transcription regulation cellular response, followed by transcription, and translation regulation. GO cellular component (CC) analysis revealed enrichment in pathways associated mainly with the nucleus and cytoplasm. In the molecular function (MF), the significant items were activation and repression transcription, and RNA-binding. The record of functional annotations of the genes related to HTLV-1 infections is summarized in Figure 8. KEGG analysis of predicted target genes from differentially expressed miRNAs in HTLV-1 and HTLV-2 asymptomatic carriers revealed significant enrichment in cancer-related pathways, signaling cascades (e.g., FoxO, Ras, MAPK), and viral infection processes, with pancreatic cancer, pathways in cancer, and EGFR tyrosine kinase inhibitor resistance as the top three enriched pathways as shown in Figure 9.

### Network analysis

**Figure 10:**
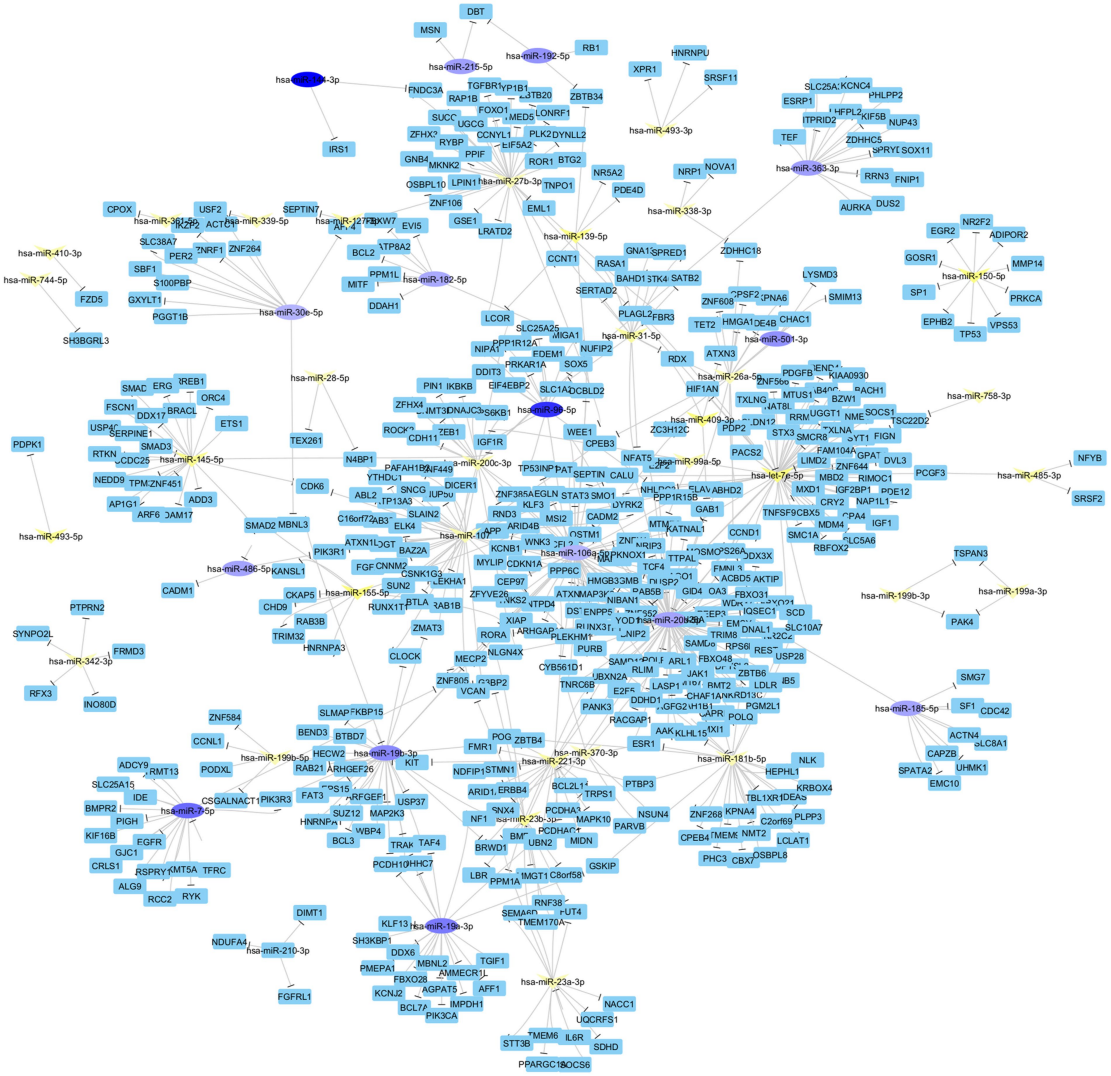
Interaction network constructed from differentially expressed miRNAs to identify key genes and transcription factors. The network consists of 542 nodes and 623 edges, averaging 2.299 neighbors per node, and features 12 connected components without multi-edge node pairs or self-loops. Key miRNAs with strong connections to target genes between the HTLV-1 group and healthy controls are highlighted.

The differentially expressed miRNAs were used for construction of an interaction network for the identification of important genes and transcription factors. The network consisted of 542 nodes and 623 edges, with an average of 2.299 neighbors per node (Figure 10). There were 12 connected components, with no multi-edge node pairs or self-loops, reflecting a straightforward interaction model. In the analysis of miRNAs with strong connections to target genes between the HTLV-1 group and HC, several key miRNAs were identified. For example, hsa-miR-20b-5p has the highest degree and betweenness, suggesting it is a central regulator with extensive interactions, with a degree of 87 and downregulated expression (Log FC −4.17) (Supplementary Figure S5). This was followed by hsa-let-7e-5p with a degree of 60 and upregulated expression (Log FC 7.08), hsa-miR-106a-5p with a degree of 58 and downregulated expression (Log FC −3.46), hsa-miR-107 with a degree of 36 and upregulated expression (Log FC 3.97), hsa-miR-27b-3p with a degree of 34 and upregulated expression (Log FC 3.78), and hsa-miR-19b-3p with a degree of 29 and downregulated expression (Log FC −5.38).

## Discussion

The main objective of this study was to investigate the effects of HTLV-2 infection on sRNA expression profiles and compare them with those of HTLV-1-infected individuals and HC to elucidate epigenetic molecular changes in these chronic infections. Through rigorous statistical analyzes, we identified 331 known sRNAs that differed significantly between the three groups, including important miRNAs. Heatmap analysis revealed a distinct clustering of sRNAs, with a notable cluster showing sRNAs that were upregulated in HC compared to HTLV-1- and HTLV-2-infected individuals. These results suggest a possible role of sRNA dysregulation in HTLV pathogenesis and its impact on host cellular functions.

Although HTLV-1 and HTLV-2 are closely related retroviruses, they differ markedly in their clinical manifestations and molecular profiles. HTLV-1 is closely associated with severe diseases such as ATLL and HAM/TSP, whereas HTLV-2 is less pathogenic and less frequently associated with malignant or neurological diseases (34). These differences may be due to differences in viral gene expression, host immune response and interaction with the host cellular system (35). Our results contribute to the growing body of evidence that sRNAs play a critical role in these virus-host interactions and may influence disease progression and immune evasion mechanisms.

We found that HTLV-1 infection resulted in upregulation of 295 sRNAs and downregulation of 47 sRNAs compared to HC. Among these, SCARNA5 was most upregulated and hsa-miR-96 was most downregulated upon HTLV-1 infection. SCARNA5, a small nuclear RNA (snoRNA), has previously been associated with poorer recurrence-free survival in estrogen receptor-positive breast cancer (36). Its high abundance in HTLV-1-infected individuals suggests a possible role in viral replication or immune modulation, which warrants further investigation. In contrast, the downregulation of hsa-miR-96, which has been implicated in neurodegenerative diseases and immune regulation, suggests a potential disruption in cellular homeostasis induced by HTLV-1 infection. These findings align with previous studies indicating miRNA deregulation as a critical factor in HTLV-1 pathogenesis (37).

In contrast, HTLV-2 infections were characterized by the upregulation of 14 sRNAs and downregulation of 44 sRNAs compared to HC, indicating unique sRNA expression patterns in HTLV-2 pathogenesis. Pilotti et al. (38) reported an HTLV-2-specific miRNA signature affecting T helper cell differentiation and cytokine pathways, emphasizing the distinct molecular mechanisms of HTLV-2 infections. While the same miRNAs were not identified in our study, our PCA analysis revealed distinct molecular signatures associated with HTLV-2 infection, supporting the notion that HTLV-2 modulates host sRNA profiles differently than HTLV-1. These observations suggest that HTLV-2-specific sRNA alterations may influence viral persistence and immune evasion.

Although the sRNAs of HTLV-1 and HTLV-2 do not overlap, some common signaling pathways have been identified. Thus, the upregulation of specific sRNAs in both groups may be due to shared host-virus interactions. This is consistent with findings in related retroviruses such as bovine leukemia virus (39), which demonstrate the influence of viral miRNAs on host transcriptional networks. Understanding these common and unique molecular interactions may provide insights into the broader mechanisms of retroviral persistence and pathogenesis.

Our analysis also detected dysregulated tRNAs in HTLV infections, specifically chr6.trna50 (downregulated) and chr11.trna16 (upregulated), which may indicate viral manipulation of the host translation machinery. Such dysregulation could represent a strategic viral adaptation to optimize replication while evading host defenses. In particular, tRNAPro, which serves as a primer for HTLV reverse transcription, could be targeted by the virus or host defenses to affect replication efficiency (40). These findings are consistent with a previous study by Mathew et al. (41), which demonstrated the role of tRNA-derived fragments (tRFs) in modulating viral infections and immune responses.

Moreover, the differential expression of mature miRNAs such as hsa-miR-20b-5p and hsa-let-7e-5p, known for their role in immune regulation and cancer (42, 43), suggests that they are involved in the maintenance of viral latency and modulation of disease progression. These miRNAs could serve as biomarkers or therapeutic targets, warranting functional validation by knockdown or overexpression studies. Furthermore, our results emphasize the importance of sRNA-mediated post-transcriptional regulation in HTLV infection, which may represent a novel target for therapeutic intervention.

Although our study highlights the potential of sRNAs as biomarkers for differentiating HTLV-1 and HTLV-2 infections, we recognize that other diagnostic methods remain the gold standard due to their efficiency and reliability. However, the unique molecular signatures identified by PCA analysis support the potential development of precision diagnostics tailored to individual sRNA profiles. These findings could contribute to improved disease monitoring and refined therapeutic interventions for HTLV-associated diseases, although further validation in larger cohorts and functional studies is required to determine their clinical utility.

The main limitations of our study include the relatively small sample size, which may limit the generalizability of our results and increase the risk of type II errors. In addition, the cross-sectional nature of our analysis limits our ability to infer causal relationships between sRNA expression changes and disease progression. Future studies should aim to validate these results in larger cohorts and perform longitudinal analyzes to capture dynamic sRNA changes over time. Functional studies are also needed to determine the exact role of the identified sRNAs in viral replication, immune modulation and disease progression.

## Conclusion

This study reveals distinct sRNA expression patterns in HTLV-1 and HTLV-2 infections, highlighting their potential as biomarkers and therapeutic targets. The differential expression of sRNAs and tRNAs provides new insights into the molecular mechanisms governing HTLV pathogenesis. While limitations such as sample size and lack of longitudinal data exist, these findings lay a foundation for future research to validate these biomarkers and explore their role in HTLV-associated diseases. Understanding the regulatory networks involving sRNAs in HTLV infections could pave the way for novel diagnostic and therapeutic strategies, ultimately improving patient management and disease outcomes.

## Data Availability

The datasets presented in this study can be found in online repositories. The names of the repository/repositories and accession number(s) can be found at: https://doi.org/10.5281/zenodo.14041731.

## Footnotes

^1^ www.cytoscape.org
