# Supplementary material for: HTLV-1 and HTLV-2 infections significantly alter small RNA expression in asymptomatic carriers
Source: Front Med (Lausanne). 2025 Feb 17;12:1547712. doi: 10.3389/fmed.2025.1547712 (PMC11872698; doi:10.3389/fmed.2025.1547712)
Supplement: Supplementary file 1 [file Data_Sheet_1.zip › Supplementary Material/Legend to Supplementary Figures.docx]

**HTLV-1 and HTLV-2 Infections Significantly Alter Small RNA Expression in Asymptomatic Carriers**

Lorena Abreu Fernandes^1^, Victor Ângelo Folgosi^2^, Rodrigo Pessoa^1^, Tatiane Assone^2^, Jefferson Russo Victor^2^, Jorge Casseb^2^, Augusto César Penalva de Oliveira^3^, Youko Nukui^4^, Alberto José da Silva Duarte^2^, Sabri Saeed Sanabani^2,5^*.

^1^Post-Graduation Program in Translational Medicine, Department of Medicine, Federal University of São Paulo, São Paulo, 04021-001, Brazil.

^2^Laboratory of Medical Investigation LIM-56, Division of Dermatology, University of São Paulo, Medical School, São Paulo, Brazil.

^3^Department of Neurology, Institute of Infectology Emílio Ribas (IIER), São Paulo, Brazil.

^4^Department of Hematology, Faculty of Medicine, University of São Paulo, São Paulo 05403-000, Brazil.

^5^Laboratory of Medical Investigation 03 (LIM03), Clinics Hospital, Faculty of Medicine, University of São Paulo, São Paulo 05403-000, Brazil.

* Corresponding author

Sabri Saeed Sanabani, PhD

E-mail: sabyem_63@yahoo.com

Laboratory of Dermatology and Immunodeficiency, LIM56/03.

Instituto de Medicina Tropical de São Paulo

Faculdade de Medicina da Universidade de São Paulo

Av. Dr. Eneas de Carvalho Aguiar, 470 3º andar

São Paulo Brazil 05403 000

Phone: + 5511 3061 7194 ext:218

**Titles for Supplementary Tables**

S1- List of significantly expressed known small RNAs across HTLV-1 infected, HTLV-2 infected, and healthy control groups.

S2- List of significantly expressed novel small RNAs across HTLV-1 infected, HTLV-2 infected, and healthy control groups.

S3- List of significantly expressed mature miRNAs across HTLV-1 infected, HTLV-2 infected, and healthy control groups.

S4- Predicted target genes of differentially expressed miRNAs identified using the GSEA algorithm in the miRWalk tool.

S5- Full record of remarkable functional annotations of target genes.

**Legend to Supplementary Figures**

1. (A) Venn diagram illustrating known small RNA (sRNA) whose expression was either upregulated or downregulated (≥10-fold) in peripheral blood mononuclear cells across four entity lists: upregulated sRNAs in the HTLV-2 group (entity list 1), downregulated in HTLV-2 (entity list 2), upregulated in the HTLV-1 group (entity list 3), and downregulated in HTLV-1 (entity list 4). (B) 3-D representation of principal component analysis (PCA) showing the distances between the three groups based on the 331 sRNA expression profiles.
2. Heatmap of novel small RNA (sRNA) expression profiles in healthy controls (HC), HTLV-2, and HTLV-1 groups. The dendrograms represent hierarchical clustering of samples and sRNA, highlighting distinct expression patterns across the groups. Red, blue, and yellow colors indicate the HC, HTLV-2, and HTLV-1 groups, respectively. The intensity of the colors in the heatmap corresponds to the level of small RNA expression, with red representing high expression and blue representing low expression.
3. Venn diagram illustrating novel small RNA (sRNA) whose expression was either upregulated or downregulated (≥10-fold) in peripheral blood mononuclear cells across four entity lists: upregulated sRNAs in the HTLV-2 group (entity list 1), downregulated in HTLV-2 (entity list 2), upregulated in the HTLV-1 group (entity list 3), and downregulated in HTLV-1 (entity list 4).
4. (A) Venn diagram illustrating mature microRNA (miRNA) whose expression was either upregulated or downregulated (≥10-fold) in peripheral blood mononuclear cells across four entity lists: upregulated sRNAs in the HTLV-2 group (entity list 1), downregulated in HTLV-2 (entity list 2), upregulated in the HTLV-1 group (entity list 3), and downregulated in HTLV-1 (entity list 4). (B) 3-D representation of principal component analysis (PCA) showing the distances between the three groups based on the 120 miRNA expression profiles.
5. Interaction network highlighting hsa-miR-20b-5p as a central regulator with the highest degree (87) and betweenness, indicating extensive interactions. This miRNA is downregulated (Log FC -4.17) and connects to multiple target genes, illustrating its key role in the regulatory network between the HTLV-1 group and healthy controls. Other significant miRNAs include hsa-let-7e-5p, hsa-miR-106a-5p, hsa-miR-107, hsa-miR-27b-3p, and hsa-miR-19b-3p, each with distinct degrees and expression changes.
