# Supplementary figures and images for: HTLV-1 and HTLV-2 infections significantly alter small RNA expression in asymptomatic carriers
Source: Front Med (Lausanne). 2025 Feb 17;12:1547712. doi: 10.3389/fmed.2025.1547712 (PMC11872698; doi:10.3389/fmed.2025.1547712)

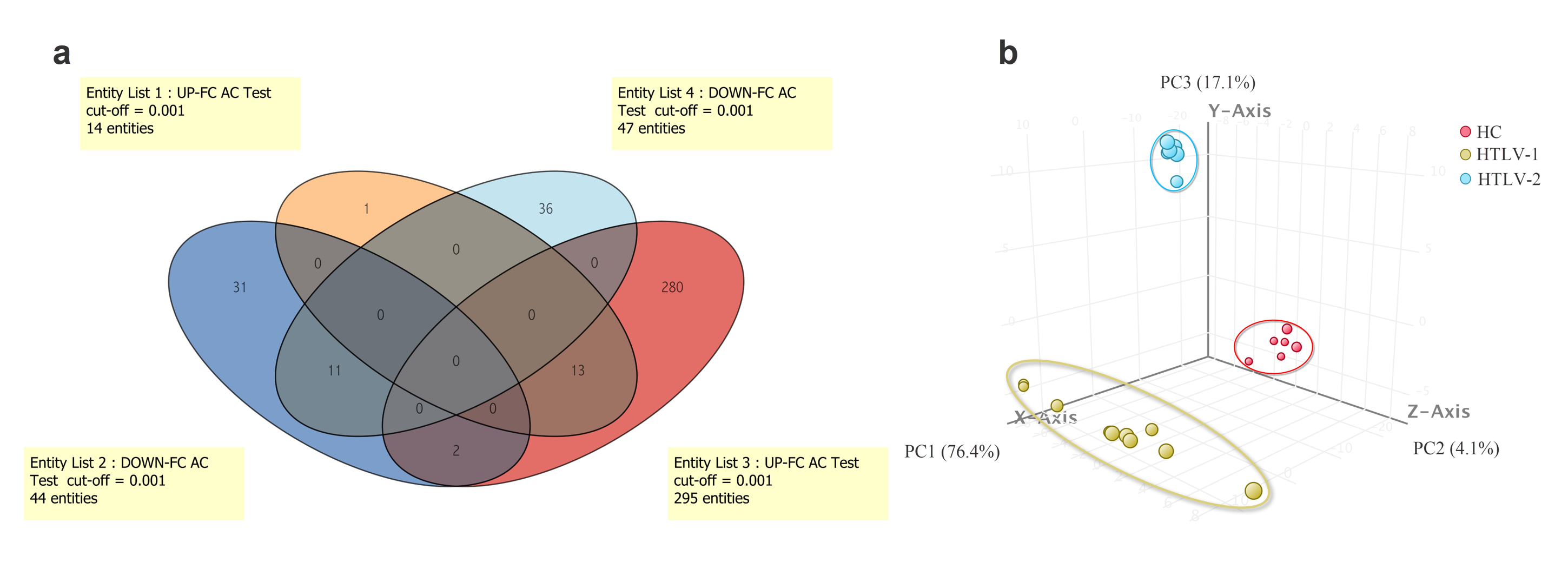

Supplement: Supplementary file 1 [file Data_Sheet_1.zip › Supplementary Material/Supplementary Figures/Figure S1.png]

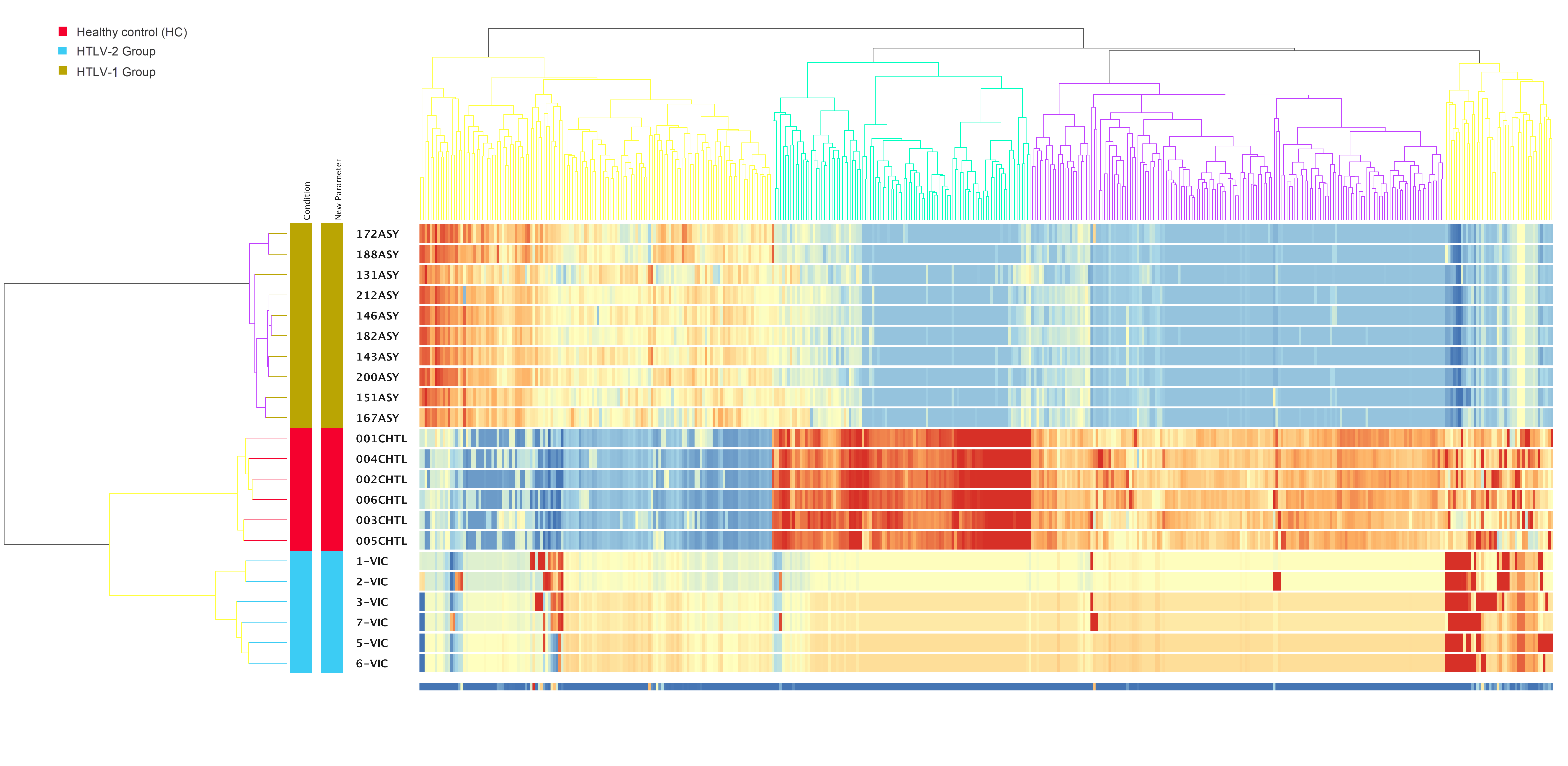

Supplement: Supplementary file 1 [file Data_Sheet_1.zip › Supplementary Material/Supplementary Figures/Figure S2.png]

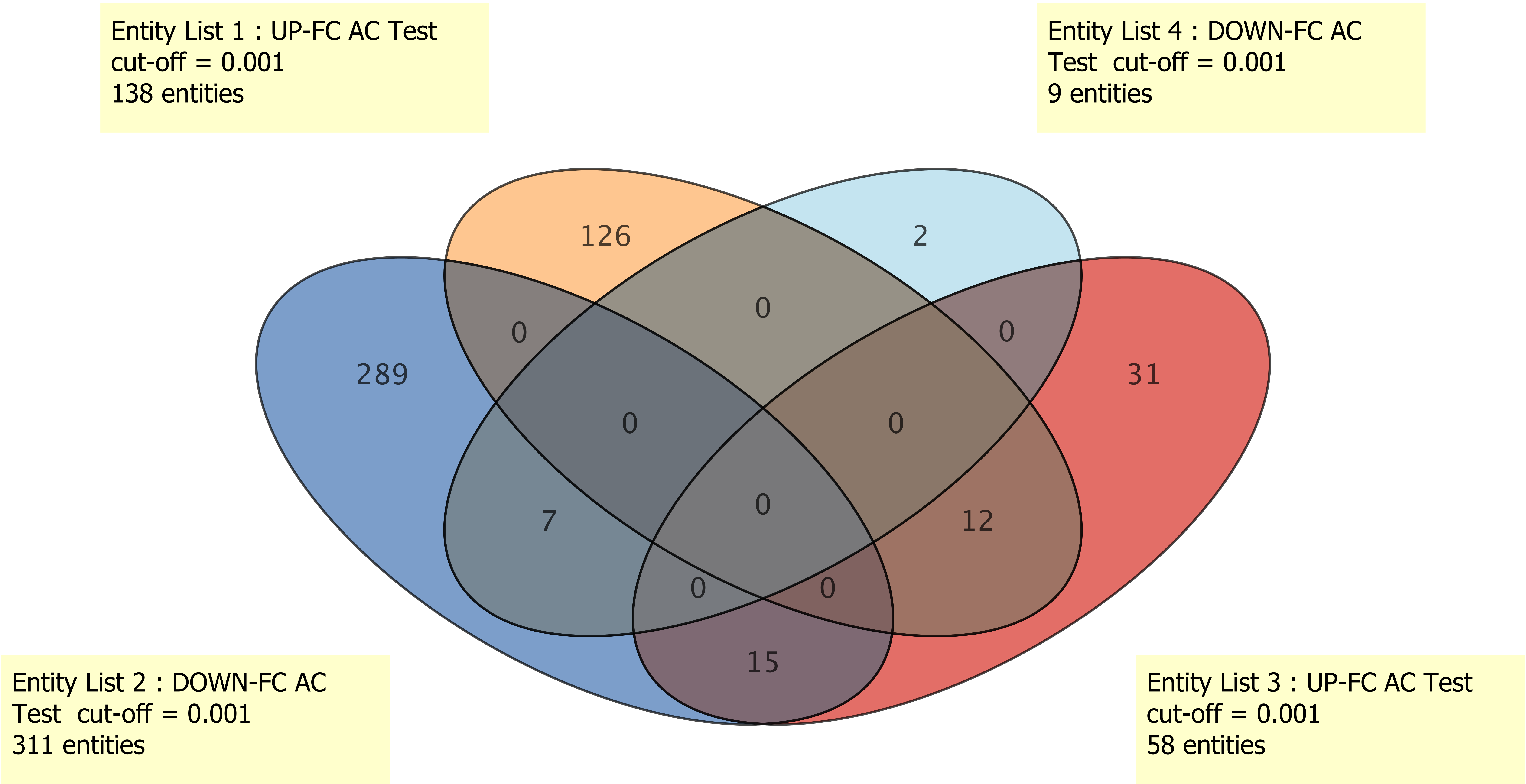

Supplement: Supplementary file 1 [file Data_Sheet_1.zip › Supplementary Material/Supplementary Figures/Figure S3.png]

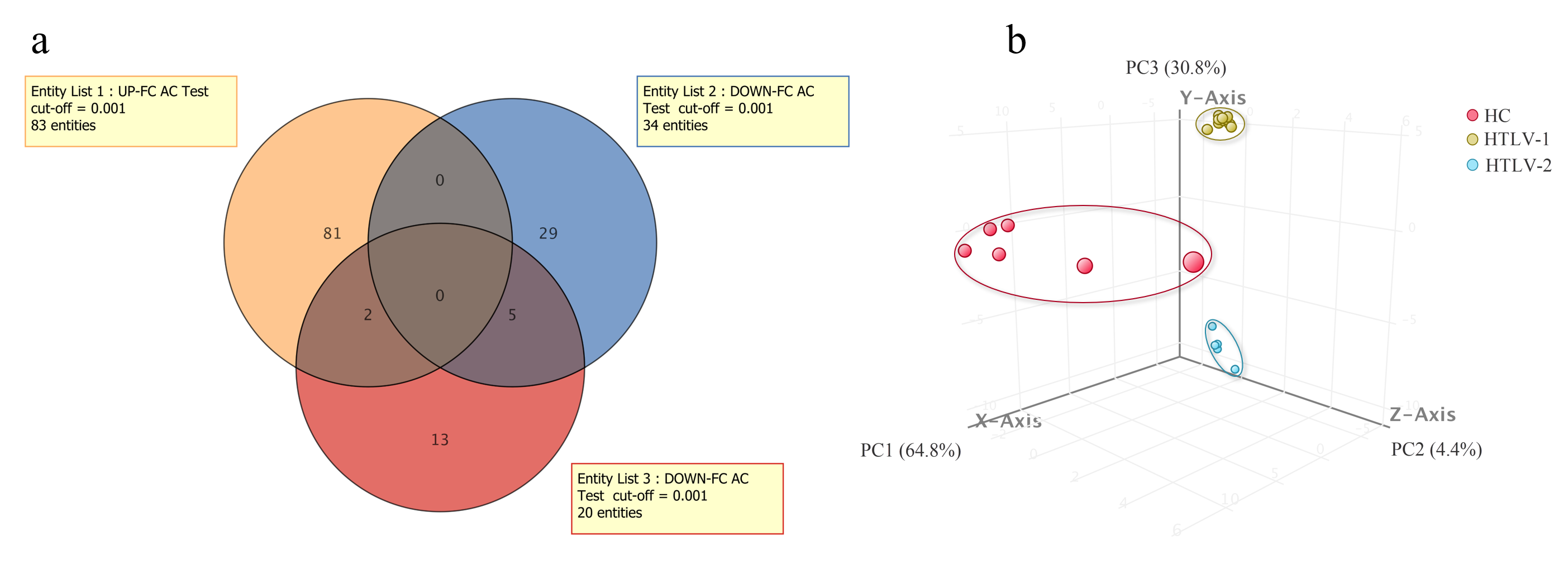

Supplement: Supplementary file 1 [file Data_Sheet_1.zip › Supplementary Material/Supplementary Figures/Figure S4 .png]

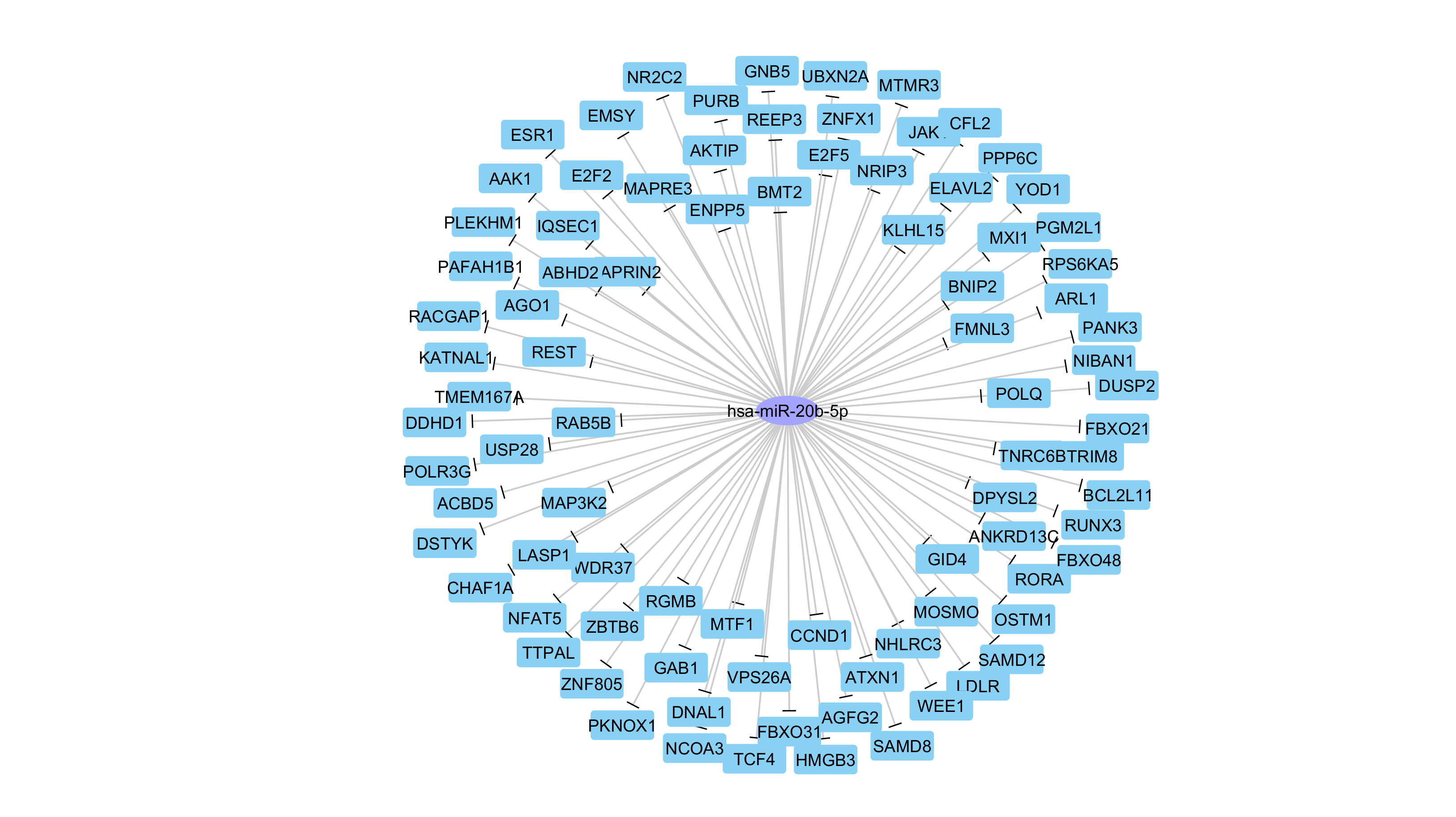

Supplement: Supplementary file 1 [file Data_Sheet_1.zip › Supplementary Material/Supplementary Figures/Figure S5.png]
